# Microarray Analysis of Paramylon, Isolated from Euglena Gracilis EOD-1, and Its Effects on Lipid Metabolism in the Ileum and Liver in Diet-Induced Obese Mice

**DOI:** 10.3390/nu13103406

**Published:** 2021-09-27

**Authors:** Seiichiro Aoe, Chiemi Yamanaka, Kento Mio

**Affiliations:** 1 The Institute of Human Culture Studies, Otsuma Women’s University, Chiyoda-ku, Tokyo 102-8357, Japan; chiemiy@gmail.com; 2 Studies in Human Life Sciences, Graduate School of Studies in Human Culture, Otsuma Women’s University, Chiyoda-ku, Tokyo 102-8357, Japan; d5120401@cst.otsuma.ac.jp

**Keywords:** paramylon, abdominal fat, DNA microarray, gene ontology, PPAR signaling

## Abstract

We previously showed that supplementation of a high fat diet with paramylon (PM) reduces the postprandial glucose rise, serum total and LDL cholesterol levels, and abdominal fat accumulation in mice. The purpose of this study was to explore the underlying mechanism of PM using microarray analysis. Male mice (C57BL/BL strain) were fed an experimental diet (50% fat energy) containing 5% PM isolated from *Euglena gracilis* EOD-1 for 12 weeks. After confirming that PM had an improving effect on lipid metabolism, we assessed ileal and hepatic mRNA expression using DNA microarray and subsequent analysis by gene ontology (GO) classification and Kyoto Encyclopedia of Genes and Genomes (KEGG) pathway analysis. The results suggested that dietary supplementation with PM resulted in decreased abdominal fat accumulation and serum LDL cholesterol concentrations via suppression of the digestion and absorption pathway in the ileum and activation of the hepatic PPAR signaling pathway. Postprandial glucose rise was reduced in mice fed PM, whereas changes in the glucose metabolism pathway were not detected in GO classification and KEGG pathway analysis. PM intake might enhance serum secretory immunoglobulin A concentrations via promotion of the immunoglobulin production pathway in the ileum.

## 1. Introduction

Obesity and overweight are worldwide problems caused by lifestyle and genetic backgrounds [1]. Abdominal fat obesity causes a pro-inflammatory status in several organs, such as the liver, adipose tissue, and the pancreas [2]. Chronic inflammation induces glucose intolerance, dyslipidemia, and an impaired immune defense system, which suggests that lifestyle changes, especially dietary habits, are very important. Dietary fiber is a beneficial food component, which is reported to improve obesity-related diseases [3]. Viscous or fermentable dietary fibers improve glucose intolerance [4], prevent body-weight gain [5], and improve dyslipidemia [6,7].

Beta-glucans are dietary fibers with very different physiological functions, depending on their origin and structure. Beta-1,3-1,4-glucan is a typical viscous and fermentable dietary fiber found in cereals; it is reported to play a crucial role in the modification of obesity-related disorders. Oat β-glucan reduces postprandial glucose increases [8,9] and serum LDL cholesterol levels in hypercholesterolemic subjects [9]. Barley β-glucan reduces the abdominal fat area [10] and improves glucose intolerance [11]. Beta-1,3-1,6-glucan, found in yeast and mushroom, is an almost insoluble dietary fiber, reported to enhance the immune response via dectin-1 [12].

Paramylon (PM) is a β-1,3-glucan, found in *Euglena gracilis* at levels of 20–70% (dry weight basis). It has been reported that PM has various effects, including an immunomodulating effect [13,14,15]. β-1,3-glucan has a triple helix structure and is classified as an insoluble dietary fiber [16]; therefore, it is considered that the physiological properties of PM differ from those of cereal β-glucan or yeast and fungal β-glucan.

There are very few reports on the effects of PM intake on metabolic disorders. Recent studies reported that PM did not have anti-obesity or anti-inflammatory effects in mice fed a high fat diet [17,18]; however, this may have been due to insufficient PM supplementation of the experimental diets [19]. We previously reported that PM had a beneficial effect in preventing obesity related parameters; in particular, abdominal fat accumulation and serum LDL cholesterol levels. We also observed an amelioration in postprandial glucose levels [20]. The mechanism underlying the anti-obesity effect of PM intake is unknown; the purpose of this study was to investigate the potential mechanism of PM modulation of lipid metabolism using DNA microarray with gene ontology (GO) classification and Kyoto Encyclopedia of Genes and Genome (KEGG) pathway analysis. It is reported that “GO characterizes the relationship between genes by specifically annotating and categorizing a gene product’s molecular function and associated biological process” [21]. The KEGG pathway is a collection of pathway maps which includes molecular interactions, reaction and relationship networks for metabolism, genetic information processing, and cellular processes [22].

## 2. Materials and Methods

### 2.1. PM Isolation and Dietary Fiber Analysis

Kobelco Eco-Solutions Co., Ltd., (Kobe, Japan) provided PM powder isolated from *Euglena gracilis* EOD-1, which is known for its high yields of PM [23]. In accordance with previously reported culture conditions, glucose was used as the carbon source [20]. Total dietary fiber was 97.8 g/100 g, determined by the AOAC 991.43 method [24].

### 2.2. Animals and Study Design

Twenty C57BL/6J mice (4 week old, male) were purchased from Charles River Laboratories Japan (Yokohama, Japan). They were maintained on a 12 h light/dark cycle from 8:00 to 20:00. Mice were individually housed in plastic carbonate cages and given ad libitum access to water. This animal experiment was approved by the Otsuma Women’s University Animal Research Committee (No. 19003, Tokyo, Japan) and was performed in accordance with the Regulation on Animal Experimentation at Otsuma Women’s University. Mice were pre-reared for 7 d before being randomly divided into two groups (*n* = 10 per group) and fed the experimental diet containing 50 g/kg cellulose (control) or 51.2 g/kg PM (corresponding to 5% dietary fiber). Dietary compositions are shown in Table 1. The experimental diets were given to mice ad libitum for 12 weeks. The food intake and body weight of each mouse were measured three times per week during the experimental period. After eight hours of fasting, blood samples were withdrawn from the heart after isoflurane/CO_2_ euthanasia. Serum was collected by centrifugation for lipid analysis. Liver, cecum with digesta, and abdominal fat (epididymal, retroperitoneal, and mesenteric fat) were dissected and weighed. Liver tissue and tissue of the ileum near the cecum (150 mg), were soaked in RNAprotect reagents (Qiagen, Hilden, Germany), and a freeze-dried residue of liver tissue was stored at −20 °C until cholesterol and triglyceride assays were performed.

### 2.3. Serum and Hepatic Biochemical Analysis

Hitachi 7180 auto-analyzers were used to analyze serum total, low-density lipoprotein (LDL), and high-density lipoprotein (HDL) cholesterols, triglycerides, non-esterified fatty acids (NEFA), and ketone body concentrations at Oriental Yeast Co., Ltd. (Shiga, Japan). Enzyme-linked immunosorbent assay (ELISA) was used to measure serum insulin (mouse insulin ELISA kit, Shibayagi Co., Ltd., Gunma, Japan) and sIgA concentrations (ELISA Kit for Secretory Immunoglobulin A, Cloud-Clone Corp., Katy, TX, USA). Hepatic cholesterol and triglyceride levels were analyzed according to a previous report [20,25].

### 2.4. Oral Glucose Tolerance Test

The experimental diets were given to mice for 12 weeks and an oral glucose tolerance test (OGTT) was performed, after 8 h fasting, during the final week. After oral glucose gavage (1.5 g/kg), a glucometer (Glutest Ace R, Sanwa Kagaku Kenkyusho Co., Ltd., Aichi, Japan) was used to measure blood glucose levels from the tail tip at 0, 15, 30, 60 and 120 min.

### 2.5. DNA Microarray Analysis

Total RNA in ileum and liver tissues were extracted using the RNeasy Mini kit (Qiagen, Hilden, Germany), according to the manufacturer’s instructions. The RNA integrity number (RIN) of all total RNA samples was analyzed and samples with values greater than 6.5 were pooled by equal mixing for each group (*n* = 10 per group) before DNA microarray analysis [26]. Procedures for microarray analysis were performed according to the manufacturer’s instructions and are described in the Supplementary File. The Gene Expression Omnibus (GEO) accession number is GSE181289.

Expression measurement annotations for upregulated and downregulated genes in each group probe were mapped to gene names using the Transcriptome Viewer (Kurabo Industries Ltd., Neyagawa, Osaka, Japan). Genes were regarded as differentially expressed when their fold change was higher than 1.3 or less than 0.77. All of the differentially expressed genes (DEGs) were characterized by their biological processes, cellular components, and molecular functions. GO enrichments were analyzed using clusterProfiler V3.6.0 (ver. 1.3.1093, R-Tools Technology Inc. (Richmond Hill, ON, Canada)). In the present study, the pathview package in R studio was used to identify and visualize the KEGG (Kyoto Encyclopedia of Genes and Genomes) pathways of the DEGs involved in lipid metabolism.

### 2.6. Expression Analysis of mRNAs Related to Lipid Metabolism in Liver and Ileum

Real-time PCR primer sequences are listed in Supplementary Table S1. mRNAs involved in liver and ileal metabolism were analyzed using SYBR^®^ Green PCR master mix (Thermo Fisher Scientific, Waltham, MA, USA) and the Applied Biosystems Quant3 real-time polymerase chain reaction (PCR) system. Data analysis was performed using the 2^−ΔΔCT^ method, where the threshold cycle (CT) indicates the fractional cycle number at which the amount of amplified target reaches a fixed threshold. The ΔCT was calculated from the difference of threshold cycles between reference gene (36B4) and target genes. The difference between the ΔCT for test diet and control diet determined the ΔΔCT. Relative expression levels are expressed as fold changes from the control group (arbitrary unit/36B4).

### 2.7. Statistical Analysis

Based on our previous measurements [20], a total of 20 mice (10 mice per group) were determined to be required in this study. Statistical data were presented as mean ± SE (standard error of the mean). Significant differences (*p* < 0.05) between group means were determined by Student’s *t*-test or Mann-Whitney U test. JMP (version 15.0, SAS Institute Inc., Cary, NC, USA) and R software were used for the statistical analyses.

## 3. Results

### 3.1. Food Intake, Body Weight and Organ Weight

Table 2 shows the growth parameters (food intake, body weight, and food efficiency ratio) in mice fed PM. The final weight and body weight gain were almost equal between the two groups. The organ weights in mice fed paramylon are shown in Table 3. Retroperitoneal and epididymal fat accumulation were lower in the PM group in comparison with the control group (*p* < 0.05). No significant differences in the weights of the liver and the cecum, and in their contents, were observed between the groups.

### 3.2. Oral Glucose Tolerance Test (OGTT)

**Figure 1:**
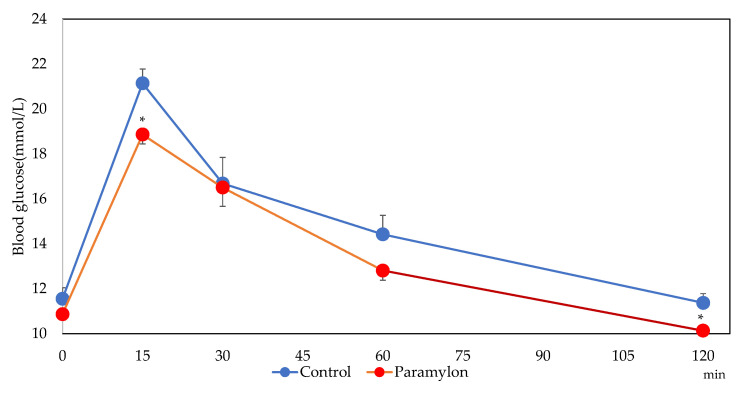
Blood glucose levels from the OGTT. Error bars represent standard error (*n* = 10). * Blood glucose levels were significantly lower at 15 min and 120 min in the PM group when compared with the control group (*p* < 0.05).

Figure 1 shows the following results of the OGTT: blood glucose levels were significantly lower at 15 min and 120 min in the PM intake group in comparison with the control (*p* < 0.05). Blood glucose levels at the other time points or in the IAUC (data not shown) were not statistically significant between the groups.

### 3.3. Biochemical Analysis of Serum and Liver Lipids

**Figure 2:**
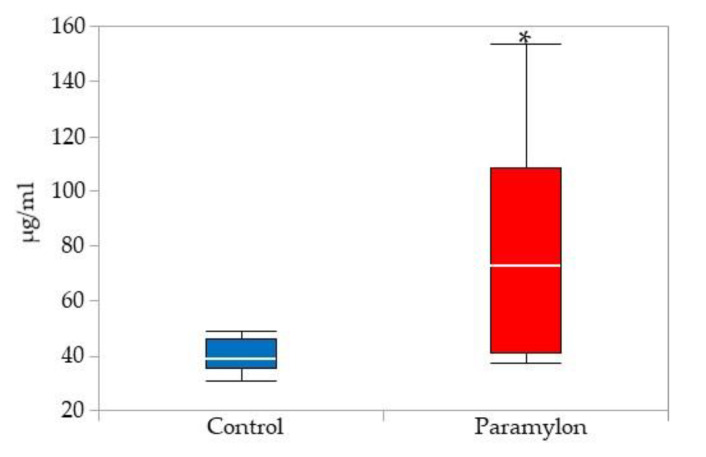
Serum sIgA concentrations. * Significantly different from the control group (Mann-Whitney test, *p* < 0.05).

Table 4 shows the serum lipid concentrations. Significant reductions in the serum total and LDL cholesterol levels were observed in the PM group in comparison with the control group (*p* < 0.05). No significant differences in other serum lipids, ketone body, and insulin concentrations were observed between the two groups. The Hepatic lipid contents are shown in Supplementary Table S2; no significant differences in cholesterol and triglyceride contents were observed between the experimental groups. The Serum sIgA concentrations are shown in Figure 2. The Serum sIgA concentrations were significantly higher in the PM group when compared with the control group (*p* < 0.05).

### 3.4. Gene Ontology (GO) and KEGG Pathway Analysis of DEGs

**Figure 3:**
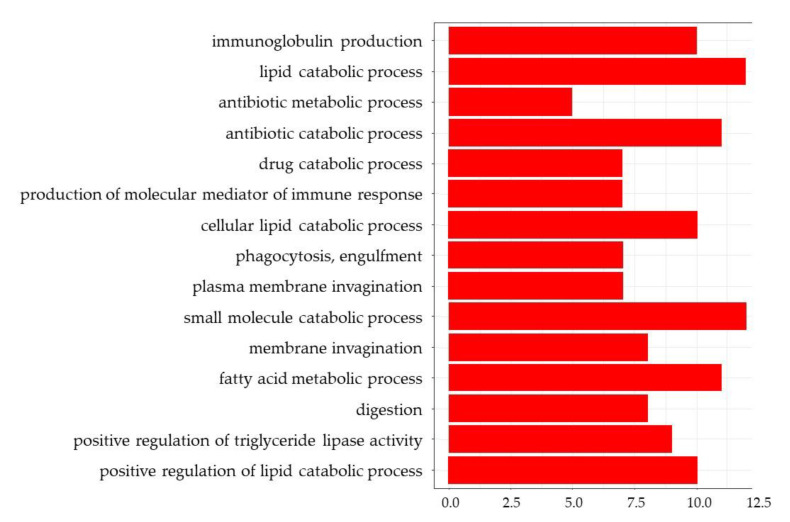
The top 15 GO terms from the Disease and Gene Annotations (DGAs) for upregulated genes in the liver.

A total of 41,345 mRNAs were screened. In the ileum, 1449 mRNAs were upregulated (>1.3 log-ratio), and 1614 mRNAs were downregulated (<0.77 log-ratio). In the liver, 1454 mRNAs were upregulated (>1.3 log-ratio), and 1452 mRNAs were downregulated (<0.77 log-ratio). GO enrichment analysis of the liver showed that the top 15 GO terms in DEGs with a greater than 1.3 (log-ratio) fold change included ‘cholesterol metabolic process’, ‘secondary alcohol metabolic process’, and ‘sterol metabolic process’ (Figure 3). No GO terms for DEGs with a less than 0.77 (log-ratio) fold change were detected in the liver.

**Figure 4:**
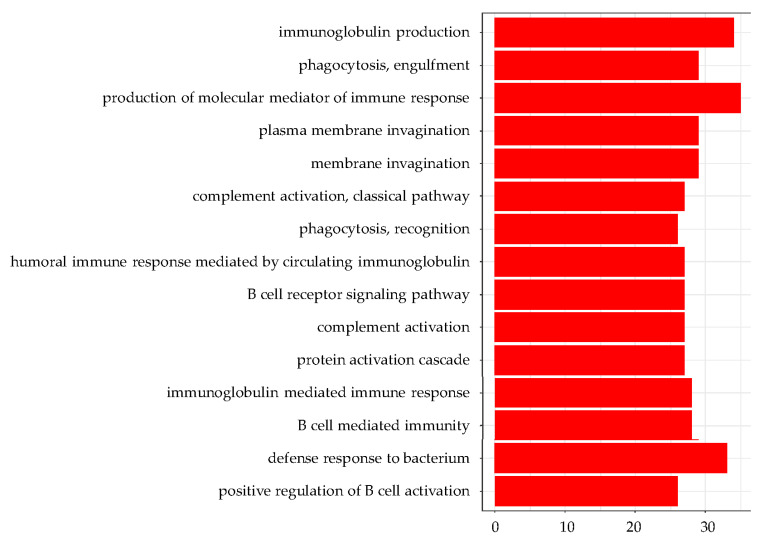
The top 15 GO terms from the DGAs for upregulated genes in the ileum.

**Figure 5:**
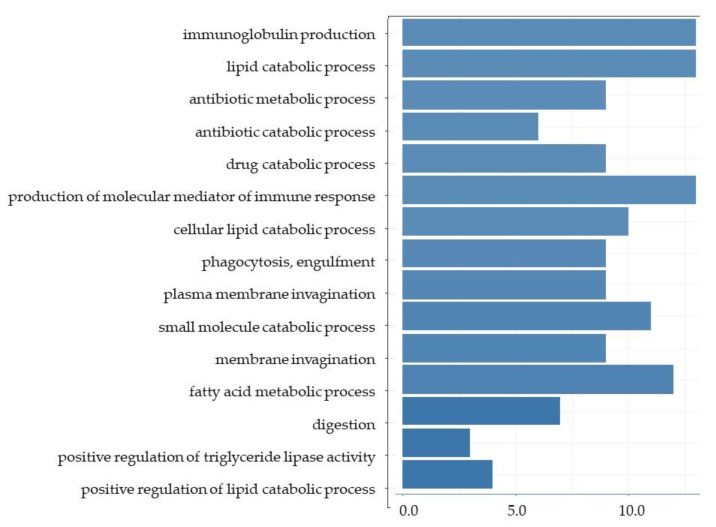
The top 15 GO terms from the DGAs for downregulated genes in the ileum.

**Figure 6:**
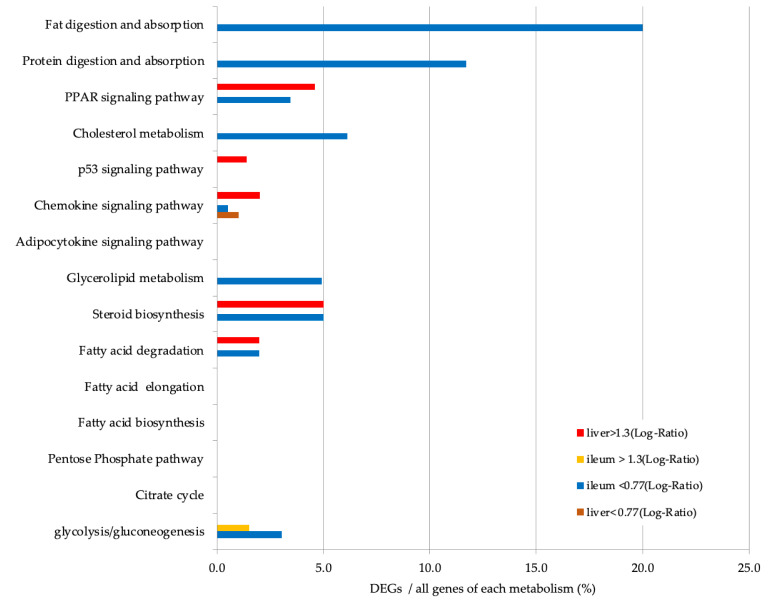
Percentage of upregulated (>1.3-fold difference to the control) and downregulated (<0.77-fold difference to the control group) differentially expressed genes (DEGs) in the liver and ileum. The y-axis shows the lipid metabolic pathway described in the Kyoto Encyclopedia of Gene and Genome (KEGG). The x-axis shows the percentage of DEGs per total genes involved in each pathway.

GO enrichment analysis of the ileum showed that almost all of the top 15 GO terms in DEGs with a greater than 1.3 (log-ratio) fold change were involved in the immune response (Figure 4). GO terms for DEGs with a less than 0.77 (log-ratio) fold change were detected in the ileum and showed that the top 15 GO terms included ‘lipid catabolic process’, ‘cellular lipid catabolic process’, ‘fatty acid metabolic process’, ‘digestion’, ‘positive regulation of triglyceride lipase activity’, and ‘positive regulation of lipid catabolic process’ (Figure 5). Furthermore, KEGG pathway analysis indicated that the upregulated pathways in the liver included the ‘PPAR signaling pathway’, ‘p53 signaling pathway’, ‘steroid biosynthesis pathway’, and ‘fatty acid degradation pathway’ (Figure 6). The downregulated pathways in the ileum included ‘fat digestion and absorption’, ‘protein digestion and absorption’, ‘PPAR signaling pathway’, ‘cholesterol metabolism’, ‘glycerolipid metabolism’, ‘steroid biosynthesis’, and ‘fatty acid degradation’.

### 3.5. mRNA Expression Levels of Genes Involved in Lipid Metabolism in the Liver and Ileum

**Figure 7:**
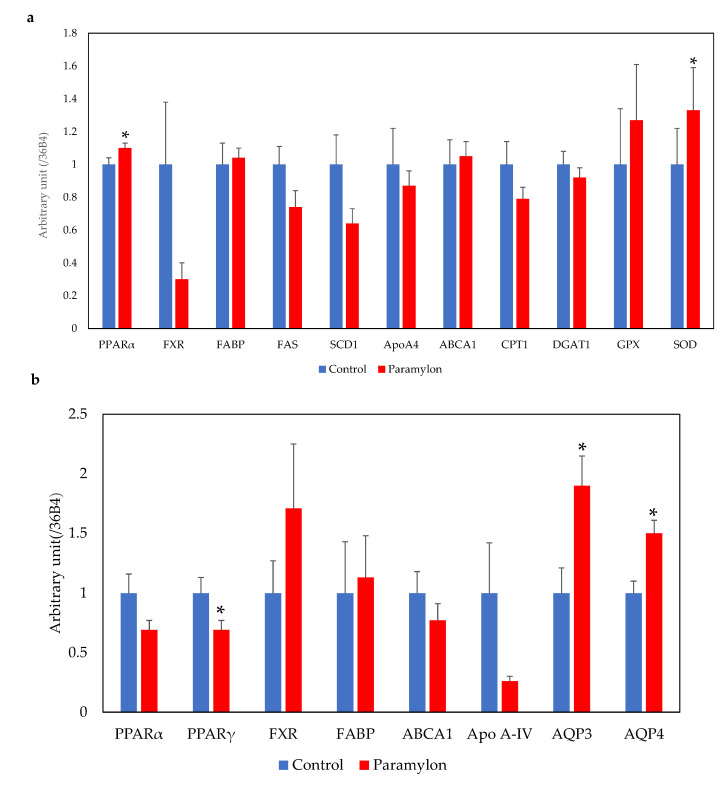
Relative expression levels of mRNAs involved in lipid metabolism in the liver (**a**) and ileum (**b**). Error bars represent standard error (*n* = 10). * Significantly different from the control group (*p* < 0.05). PPARα, peroxisome proliferator-activated receptor α; PPARγ, peroxisome proliferator-activated receptor γ; FXR, farnesoid X receptor; SCD1, stearoyl-CoA desaturase 1; Apo A-IV, apolipoprotein A-IV; ABCA1, ATP-binding cassette transporter A1; CPT1, carnitine palmitoyltransferase 1; DGAT1, diacylglycerol O-acyltransferase 1; GPX, glutathione peroxidase; SOD, superoxide dismutase; AQP 3,4, aquaporin 3,4.

mRNA expression levels in the liver (a) and ileum (b) are shown in Figure 7. PPARα mRNA expression levels were significantly higher in the livers of mice in the PM group when compared with mice in the control group (*p* < 0.05; Figure 7a). SOD mRNA expression levels were significantly higher in the PM group in comparison with the control group (*p* < 0.05). No significant differences in the mRNA expression levels of the other genes analyzed were observed in the liver. PPARγ mRNA expression levels in the ileum were significantly lower in the PM group in comparison with the control group (*p* < 0.05; Figure 7b). The mRNA expression levels of AQP3 and AQP4 were significantly higher in the PM group, in comparison with the control group (*p* < 0.05). No significant differences in the mRNA expression levels of the other genes analyzed were observed in the ileum.

## 4. Discussion

### 4.1. Fat Digestion and Absorption Pathway in the Ileum

The purpose of this study was to explore the potential mechanism of lipid metabolism modulation by PM, using GO classification and KEGG pathway analysis. Our previous report showed that PM intake reduced postprandial glucose levels and serum LDL cholesterol, and increased serum sIgA concentrations in mice, when compared with control mice [20]. The food efficiency ratio calculated from body-weight gain and food intake tended to be lower in the PM group (statistically not significant), which is similar to our previous report [20]. In addition, although no significant difference was only observed in mesenteric fat weight, the total abdominal fat weight (epididymal, retroperitoneal, and mesenteric fat) was significantly decreased in the PM group (data not shown). We confirmed that PM improved lipid metabolism in mice fed a high-fat diet, which was consistent with our previous report [20], before continuing with microarray analysis. This is the first report analyzing mRNA expression in the ileum, after PM intake. Interactions between indigestible substances, such as dietary fiber and intestinal cells, are more likely to occur in the ileum than the jejunum. In addition, the ileum contains both bile acid transporters and many L cells, which contain several receptors. The ileum is also affected by digesta. Fold changes of >1.3 and <0.77 were adopted in the microarray analysis, which is a range that has previously been used [27,28,29]. GO classification and KEGG pathway analysis consistently indicated that fat digestion and absorption was a key downregulated pathway in the ileum. Our previous report suggested that PM intake did not inhibit dietary fat absorption and did not affect microbiota constitution [20]; therefore, PM might modify the absorption process, and then inhibit chylomicron secretion. KEGG analysis of the fat digestion and absorption pathway suggested that PM suppressed the expression levels of scavenger receptor B1, FABP, ACAT, Apo A-IV, and ABCA1. A reduction in chylomicron secretion is a possible mechanism in reducing LDL cholesterol and abdominal fat accumulation. This is a new hypothesis, showing a reduction in the fat digestion and absorption pathway in the digestive tract after PM intake. However, serum lipid analysis in the chylomicron fraction was not determined. Further studies are needed to prove this hypothesis regarding chylomicron metabolism in the small intestine. We intend to investigate the influence of PM intake on chylomicron metabolism in future studies.

### 4.2. PPAR Signaling Pathway in the Ileum

KEGG pathway analysis showed downregulation of the PPAR signaling pathway in the ileum. A significant reduction in the expression of PPARγ mRNA in the ileum was observed in the PM group, in comparison with the control group (*p* < 0.05), and the same tendency was observed for PPARα (not statistically significant). PPARγ is a nuclear receptor that is involved in regulating lipid metabolism under high-fat diet conditions [30]. It is activated by fatty acids and their derivatives, and creates a lipid signaling network in the intestines, which are prone to inflammation when lipid metabolism is disturbed under high-fat diet conditions [31]. The expression of PPARs might be reduced through the reduction in fatty acid absorption; however, the expression levels of genes regulated by PPARs were not significantly altered. The data suggest that the contribution of PPARs on lipid metabolism in the ileum might be small.

### 4.3. Water Absorption Pathway in the Ileum

KEGG pathway analysis also showed upregulation of the water reabsorption pathway after PM intake. The expression levels of AQP3 and AQP4 mRNA were significantly increased in the PM group compared to the control group (*p* < 0.05), but that of APQ2 was not (data not shown). AQP3 and AQP4 have been shown to be expressed in the gastrointestinal tract [32], and preliminary data suggested that aquaporin has a role in dietary fat processing [32]. The expression of aquaporins in the intestinal tract suggests that they are involved in the transport of intestinal fluid; however, there are very few studies reporting on their functional significance. Further studies are needed to elucidate that PM may affect intestinal epithelial cells and modify the function of the gastrointestinal tract, through AQPs.

### 4.4. Immunoglobulin Production

GO classification analysis showed many enhanced GO terms for immunoglobulin production in the ileum and liver. KEGG pathway analysis highlighted ‘TNF receptor superfamily member 13C (BAFFR)’, ‘major histocompatibility complex, class II (MHC class II)’, and ‘CC motif chemokine ligand 28 (CCL28)’, suggesting an upregulated intestinal immune network for IgA production. Serum sIgA concentrations were elevated by PM intake, which was consistent with our previous report [20]. PM may have an activating effect on intestinal epithelial cells, promoting the secretion of slgA [33]. A human study, involving the intake of *Euglena* *gracilis* EOD-1 biomass that was rich in PM, reported the production of PM-specific IgA antibodies and increased salivary IgA antibody titers [34]. Our data are in agreement with these previous reports. Further studies on the analysis of protein expression levels and intracellular signaling are needed to prove the activation of the IgA production pathway in the ileum.

### 4.5. PPAR Signaling Pathway in the Liver

GO classification analysis in the liver showed that the upregulated GO terms included ‘cholesterol metabolic process’, ‘secondary alcohol metabolic process’, and ‘sterol metabolic process’. KEGG pathway analysis showed that the upregulated pathways included the ‘PPAR signaling pathway’, ‘steroid biosynthesis pathway’, and ‘fatty acid degradation pathway’. Hepatic PPARα mRNA expression was significantly increased in the PM group, when compared with the control group (*p* < 0.05). It is speculated that the mRNA expression levels of ileal PPARs were decreased by a reduction in intestinal fatty acid absorption, whereas an increase in hepatic PPARα mRNA expression might be caused by an increase in abdominal fat degradation during the fasting period. The activation of PPARα might be a key pathway in hepatic metabolism, affecting downstream mRNA in the lipid catabolic process. We previously showed that PM intake increased hepatic PPARα mRNA expression and the subsequent induction of β-oxidation, through activation of ACOX, CPT and FATP2 mRNA expression [20]. We speculated that changes in fatty acid metabolism, through upregulation of the PPAR signaling pathway, may improve lipid and glucose metabolism. In this study, no significant differences in the expression levels of genes involved in lipid metabolism were detected between the PM and control groups; however, correlations were observed between PPARα mRNA and mRNA from genes involved in the pathway downstream of PPARα. It is well documented that PPARα exhibits significant anti-inflammatory properties [35]. The hepatic mRNA expression levels of SOD were significantly increased in the PM group, compared with the control group; GPX expression levels were also increased (not statistically significant). Therefore, upregulation of hepatic PPARα expression after PM intake plays a key role in anti-inflammation and modifying lipid metabolism.

### 4.6. Glucose Tolerance

PM reduced blood glucose levels at 15 min and 120 min in the OGTT, whereas PM reduced blood glucose levels at 60 min in the previous report [20]. The peak postprandial blood glucose rise was higher in this study than in the previous report, and the fasting serum insulin concentrations were also higher in this study than in the previous report. Therefore, it is possible that the experimental conditions of mice in this study were more sensitive to detecting a significant difference in the OGTT. Significant differences in serum insulin concentrations between groups were not detected in this study or the previous study. The previous study also showed that the mRNA expression of SREBP1c, controlled by insulin concentrations, was not affected by PM intake. Therefore, the contribution of serum insulin concentrations on lipid metabolism, by PM intake, may be small. However, since the insulin concentrations in the OGTT were not measured, and the insulin tolerance test was not performed, it was not possible to clarify how paramylon affected insulin sensitivity or resistance.

A major limitation of this study was that the possible mechanism for lipid metabolism was only speculated by mRNA expressions. A second limitation was that insulin concentrations were not measured during the OGTT test, to assess insulin resistance. Future studies are needed to elucidate the mechanism for glucose and lipid metabolism, by exploring the protein expression levels and insulin secretion.

## 5. Conclusions

In conclusion, PM intake reduced serum total and LDL cholesterol levels, and abdominal fat accumulation via suppression of the digestion and absorption pathway in the ileum, and activation of the hepatic PPAR signaling pathway. Changes in the glucose metabolism pathway, by PM intake, were not detected in the GO classification and KEGG pathway analysis. PM intake might enhance sIgA concentrations via promotion of the immunoglobulin production pathway in the ileum. However, key molecules, such as endogenous ligands, to upregulate hepatic PPARα expression and intracellular signaling related to immunoglobulin production, were not determined. Further investigation is required to elucidate the molecular mechanism behind the improvement in lipid metabolism by PM intake. The improvement in lipid metabolism and enhanced gastrointestinal immune function suggests that PM intake may be useful in people who are sensitive to intestinal fermentation, such as those with gut fermentation syndrome.

## Figures and Tables

**Table 1:** Test diet compositions (g/kg).

	Control	Paramylon
Corn starch	197.5	196.3
Dextrinized corn starch	132	132
Casein	200	200
Sucrose	100	100
Soybean oil	70	70
Lard	200	200
Cellulose	50	-
Paramylon	-	51.2
AIN-93G mineral mixture	35	35
AIN-93 vitamin mixture	10	10
L-cystine	3	3
Choline bitartrate	2.5	2.5
t-butylhydroquinone	0.014	0.014

**Table 2:** Body weight gain, food intake, food efficiency ratio.

	Control	Paramylon
Initial weight (g)	20.7 ± 0.2	20.7 ± 0.2
Final weight (g)	43.4 ± 0.7	42.3 ± 0.8
Body weight gain(g/day)	0.27 ± 0.01	0.25 ± 0.01
Food intake (g/day)	2.83 ± 0.03	2.85 ± 0.03
Food efficiency ratio (%) *	9.42 ± 0.19	8.93 ± 0.28

Values are means ± SE; * food efficiency ratio = body weight gain/food intake × 100.

**Table 3:** Weight of organs.

	Control	Paramylon
Liver (g)	1.47 ± 0.05	1.42 ± 0.05
Cecum with digesta (g)	0.27 ± 0.02	0.30 ± 0.03
Retroperitoneal fat (g)	0.98 ± 0.07	0.77 ± 0.09 *
Epididymal fat (g)	2.49 ± 0.06	2.33 ± 0.06 *
Mesenteric fat (g)	0.83 ± 0.07	0.73 ± 0.07

Values are means ± SE; * significantly different from the control group (*p* < 0.05).

**Table 4:** Serum lipid and insulin concentrations.

	Control	Paramylon
Total cholesterol (mmol/L)	5.06 ± 0.11	4.60 ± 0.16 *
LDL-cholesterol (mmol/L)	0.27 ± 0.02	0.21 ± 0.01 *
HDL-cholesterol (mmol/L)	2.17 ± 0.03	2.10 ± 0.04
Triglyceride (mmol/L)	0.76 ± 0.07	0.67 ± 0.04
NEFA (μmol/L)	536.9 ± 19.3	675.3 ± 26.2
Ketone body (μmol/L)	296.4 ± 44.6	330.6 ± 59.8
Insulin (ng/mL)	3.5 ± 0.6	2.2 ± 0.4

Values are means ± SE; * Significantly different from the control group (*p* < 0.05).

## Supplementary Materials

The following are available online at www.mdpi.com/2072-6643/13/10/3406/s1. Supplementary Materials and Methods microarray analysis; Table S1: primers used for real-time reverse transcription polymerase chain reaction; Supplementary Table S2: liver lipid levels.
