# Supplementary material for: Microarray Analysis of Paramylon, Isolated from Euglena Gracilis EOD-1, and Its Effects on Lipid Metabolism in the Ileum and Liver in Diet-Induced Obese Mice
Source: Nutrients. 2021 Sep 27;13(10):3406. doi: 10.3390/nu13103406 (PMC8538961; doi:10.3390/nu13103406)
Supplement: Supplementary file 1 [file nutrients-13-03406-s001.zip › nutrients-1342208-supplementary.pdf]

2. Materials and Methods

2.5 DNA microarray analysis

The RNA integrity number (RIN) of all total RNA samples was obtained after a visual inspection of the Bioanalyzer electropherograms (Agilent Technologies Japan, Ltd., Tokyo, Japan). Samples with RIN values greater than 6.5 were used in the analysis, according to a previous report [26]. RNA was pooled by equal mixing of samples for each group (n = 10 per group) before DNA microarray analysis. Total RNA (100 ng) on the GeneChip WT PLUS Reagent Kit (Thermo Fisher Scientific, Inc., Waltham, MA, USA) was used for the microarray, according to the manufacturer's instructions. After fragmentation, samples were labelled with biotin, and the resultant single-strand cDNA was hybridized to the GeneArray Mouse 2.0ST Array using the Affymetrix 450 Fluidics Station and GeneChip Scanner 3000 7G, according to the manufacturer's instructions (Thermo Fisher Scientific, Inc., Waltham, MA, USA). Expression Console software, version 1.3 (Thermo Fisher Scientific, Inc.) with default robust multichip analysis parameters was used in the generation of expression values. The analyzes were performed by Kurabo Industries Ltd. (Osaka, Japan). Raw and standardized (logarithmic transformation) microarray data were registered in the Gene Expression Omnibus (GEO) at the National Center for Biotechnology Information (NCBI).

Table S1. Primers used in the real-time reverse transcription polymerase chain reaction.

	**Forward**	**Reverse**
**PPARα**	5'-AGGAAGCCGTTCTGTGACAT-3'	5'-AATCCCCTCCTGCAACTTCT-3'
**PPARγ**	5'-TGTCGGTTTCAGAAGTGCCTTG-3'	5'-TTCAGCTGGTCGATATCACTGGAG-3'
**FXR**	5'-ACAGAACAAGTGGCAGGTC-3'	5'-CTGAAGAAACCTTTACACCCCTC-3'
**FABP**	5'-GACCTCATCCAGAAAGGGAAG-3'	5'-TTTTTCCCCAGTCATGGTCTC-3'
**FAS**	5'-CCTGGATAGCATTCCGAACCT-3'	5'-AGCACATCTCGAAGGCTACACA-3'
**SCD1**	5'-CCTTATACATTGCCAACACCAT-3'	5'-AGCCAACCCACGTGAGAGAA-3'
**Apo A-IV**	5'-CAGTGAGGAGCCCAGGATGTT-3'	5'-TCTACAGCCTCCTTGGCATT-3'
**ABCA1**	5'-CCTCAGCCATGACCTGCCTTGTAG-3'	5'-CCGAGGAAGACGTGGACACCTTC-3'
**CPT1**	5'-GATGGAGAGGATGTTCAACACTACAC-3'	5'-AGCCCTCATAGAGCCAGACCTT-3'
**DGAT1**	5'-GCTGAGTCTGTCACCTACTTTTGG-3'	5'-AGGCTTGTAGAAGTGTCTGATGCA-3'
**GPX**	5'-GGGCAAGGTGCTGCTCATTG-3'	5'-AGAGCGGGTGAGCCTTCTCA-3'
**SOD**	5'-CAGCATGGGTTCCACGTCCA-3'	5'-CACATTGGCCACACCGTCCT-3'
**AQP3**	5'-GCTTTTGGCTTCGCTGTCAC-3'	5'-TAGATGGGCAGCTTGATCCAG-3'
**AQP4**	5'-GAGTCACCACGGTTCATGGA-3'	5'-CGTTTGGAATCACAGCTGGC-3'
**Reference 36B4**	5'-GGCCCTGCACTCTCGCTTTC-3'	5'-TGCCAGGACGCGCTTGT-3'

PPARα, peroxisome proliferator-activated receptor α; PPARγ, peroxisome proliferator-activated receptor γ; FXR, farnesoid X receptor; SCD1, stearoyl-CoA desaturase 1; Apo A-IV, apolipoprotein A-IV; ABCA1, ATP-binding cassette transporter A1; CPT1, carnitine palmitoyltransferase 1; DGAT1, diacylglycerol O-acyltransferase 1; GPX, glutathione peroxidase; SOD, superoxide dismutase; AQP 3,4, aquaporin 3,4; 36B4, acidic ribosomal protein.

Table S2. Liver lipid levels.

	Control	Paramylon
Cholesterol (μmol/liver)	19.2±1.9	20.5±0.9
(μmol /g liver)	12.8±0.9	14.4±0.2
Triglyceride (μmol /liver)	128.2±14.7	117.4±16.6
(μmol /g liver)	85.3±7.6	79.9±8.5

Values are means±SE, n=10.
